# Intraoperative Repair of Bronchial Damage Following Robotic Segmentectomy

**DOI:** 10.1111/1759-7714.70121

**Published:** 2025-07-09

**Authors:** Alfonso Fiorelli, Vincenzo Di Filippo, Beatrice Leonardi, Noemi Giorgiano, Giovanni Liguori, Francesca Capasso

**Affiliations:** ^1^ Thoracic Surgery Unit University of Campania “Luigi Vanvitelli” Naples Italy; ^2^ Anesthesiology Unit Cardarelli Hospital Naples Italy

**Keywords:** bronchial fistula, robotic surgery, segmentectomy

## Abstract

Herein, we reported the damage of the B6 bronchial segment following apical segmentectomy of the left lower lobe for management of a typical carcinoid tumor. The defect was localized distally to the B6 origin and was successfully repaired by re‐stapling the proximal side of the B6 bronchus. Before firing, the intraoperative bronchoscopy confirmed the closure of the B6 bronchus alone and the normal patency of the bronchial pyramid basal. Then, the bronchial stump was covered by a collagen patch to reduce the risk of fistula. The postoperative course was uneventful, and the patient was discharged 3 days later.

## Graphical abstract

During robotic surgery, restapling the bronchial stump may be easier than closing the bronchial defect using robotic needle drivers.

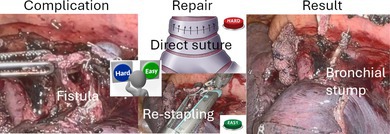

## 1 Introduction

The case highlighted a rare but critical intraoperative complication during robotic segmentectomy, namely segmental bronchial injury. We provided a pragmatic, stepwise approach to managing such complications.

## 2 Case Description

**FIGURE 1:**
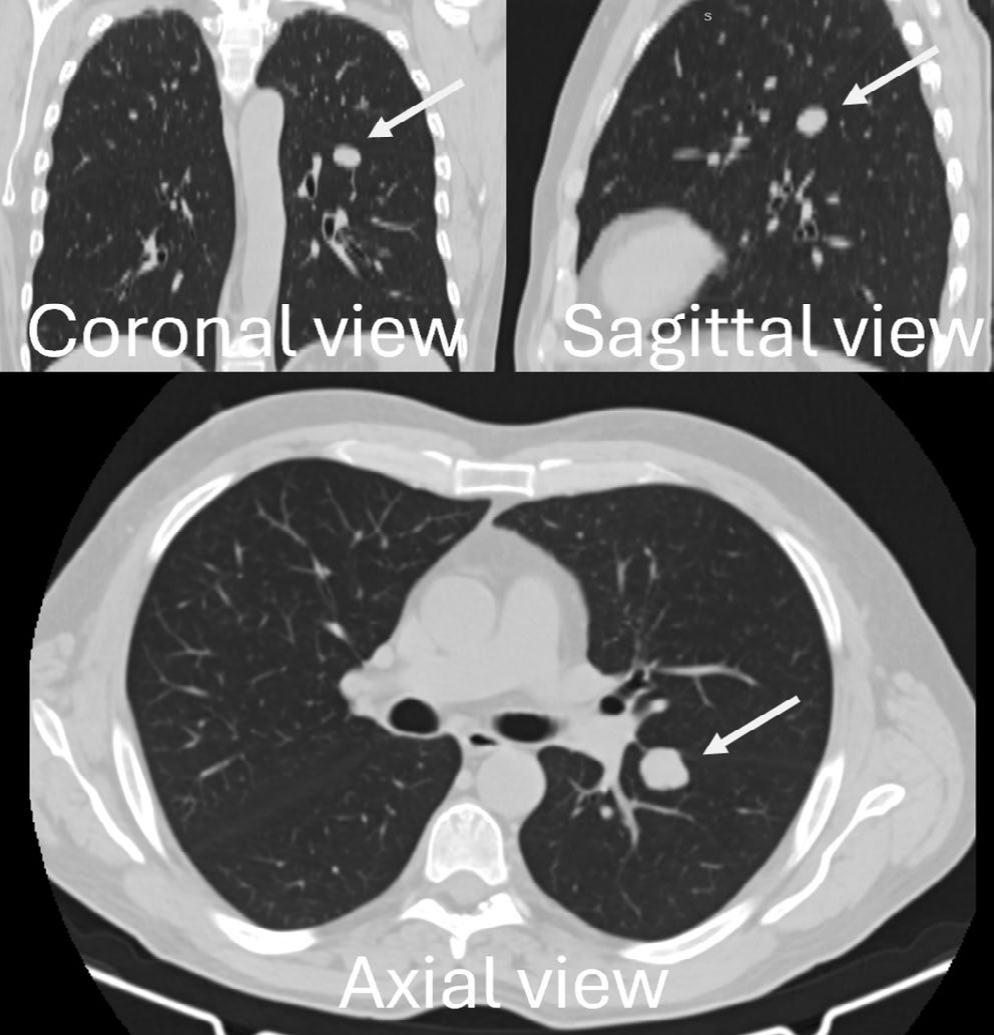
Multiplanar reconstruction of chest computed tomography scan showed a 20 mm nodule (white arrow) localized within superior segment of the left lower lobe.

A 73‐year‐old man was admitted to our hospital for management of lung typical carcinoid. Whole body Computed Tomography (CT) scan with contrast (Figure 1) and 18 FDG PET scan showed a 20 mm tumor localized within the superior segment (S6) of the left lower lobe, FDG avid (SUV uptake 4.7) with no other pathological lesion (clinical stage: T1cN0M0). CT‐guided transthoracic fine needle aspiration biopsy (FNAB) diagnosed a typical carcinoid. After a multidisciplinary team (MDT) meeting, the patient was scheduled for surgical treatment. The patient presented a limited respiratory function with a predicted postoperative (ppo) FEV1 of 41% (1.25 L) and a ppoDLCO of 45%. Thus, he was deemed unfit for lobectomy and scheduled for sublobar resections. Wedge resection was technically unfeasible as the tumor was near the fissure, and segmentectomy resulted to be the best strategy.

**FIGURE 2:**
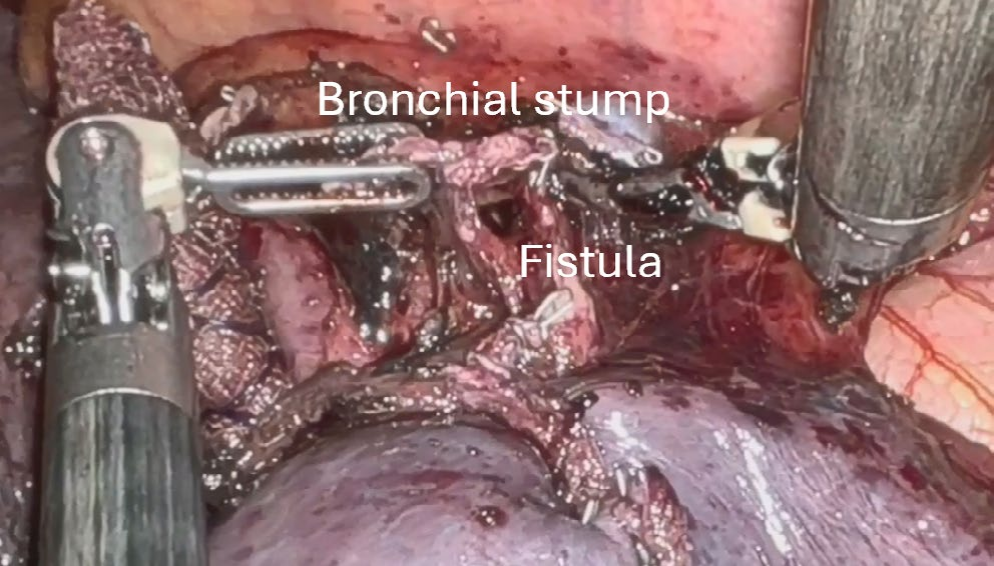
Bronchial defect, distally to the B6 origin.

**FIGURE 3:**
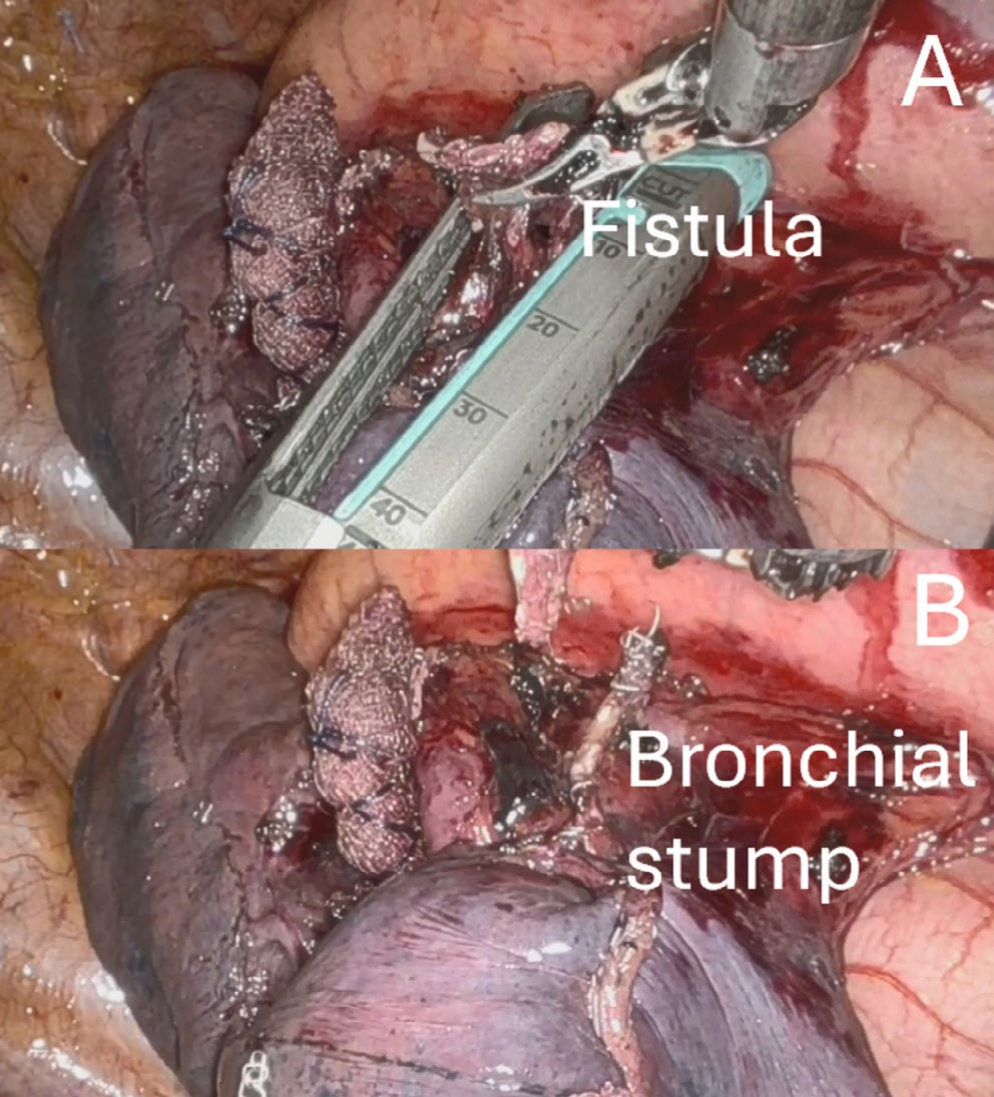
Stapler positioned proximally to the defect (A). New bronchial stump completely closed (B).

A standard robotic left four arms approach was performed. The inferior pulmonary ligament was resected; the dissection was performed in the fissure to identify and resect the arterial branch A6; the mediastinal pleura was posteriorly dissected to expose and resect the venous branch V6; then the bronchial branch B6 was isolated and resected. The parenchyma was stapled in the line of the bronchial stump, and the specimen was retrieved. Before the water test, we checked the bronchial stump and found a lesion distally to the B6 origin (Figure 2). The bronchial stump was grasped and retracted to obtain complete exposure of the defect; the stapler was positioned proximally, close to the origin of the B6 bronchus (Figure 3A) that was then stapled (Figure 3B). After closing the stapler and before firing, a flexible bronchoscopy was introduced through the double‐lumen tube, confirming the closure of the B6 bronchus alone and the normal patency of the pyramid basal bronchus. The new bronchial stump was reinforced by a collagen patch (TachoSil, Corza Medical, Austria), and no air leaks were observed after lung reinflation. Finally, radical lymphadenectomy completed the procedure, and a chest drainage was inserted in the pleural cavity through the camera port. The postoperative course was uneventful, and the patient was discharged 3 days later. At the three‐month follow‐up, no recurrence or other complications were observed. Video S1 summarized the procedure.

The authors obtained signed authorization from the patient as consent to publish the information disclosed in the study. The clinical data were available on request due to privacy.

## 3 Discussion

Intentional segmentectomy is increased use for management of early‐stage lung cancer or as an alternative to standard lobectomy in patients with limited respiratory function as in the present [1, 2, 3]. Thoracoscopic segmentectomy is associated with a reduction of complication rates compared to open surgery, but it remains a challenging procedure. Robotic technology overcomes the main limitations of thoracoscopy as the restricted movements due to the rigid instruments, the fulcrum effect, and the tremor, facilitating segmentectomy. Nevertheless, robotic lung resections including segmentectomy may be associated with unexpected intraoperative complications as hemorrhage due to vascular lesions, and injury to the airway, and/or to the abdominal organs [4, 5].

In this case, the B6 bronchus did not offer resistance during the isolation by robotic forceps nor during stapler placement. For that, the lesion remained misdiagnosed during resection, and it was evident only after specimen retrieval, when the bronchial stump was checked before the water test. In theory, the B6 bronchus was isolated distally from its origin, resulting in the damage of subsegmental branches, and the stapler was then placed not centrally. Thus, the defect remained on the distal side of the initially stapled bronchial stump and was not evident after resection.

Direct repair was considered the only feasible strategy as the patient did not tolerate more extended resections as lobectomy. In similar cases, other authors [3, 4] closed the defect of the bronchial stump using robotic needle drivers without thoracotomy conversion. However, in this case, the defect was localized at the level of the origin of the B6 bronchus, making direct suture by barbed stitches particularly challenging for surgeons with limited experience in robotic lung resections. Furthermore, the traction of the B6 bronchial stump during the maneuvers of repair could result in a larger defect, needing more complex broncho‐plastic procedures to manage it. Thus, re‐stapling the proximal side of the injured B6 bronchus seemed to be the simpler strategy to repair the fistula. Before firing, the intraoperative bronchoscopy confirmed the closure of the B6 bronchus alone and the normal patency of the bronchial pyramid basal. Then, the bronchial stump was covered by a collagen patch to reinforce the suture and reduce the risk of fistula.

Our strategy may be useful for thoracic surgeons, particularly those with limited experience in robotic lung resections. However, it should be reserved for selected circumstances as isolated segmental bronchial lesions, while bronchoplastic procedures are needed to repair more extended defects.

## Author Contributions

A.F.: write and conceptualization. V.D.F., B.L., N.G., G.L., F.C.: data and figures collection.

## Consent

An informed written consent was obtained by the patient for the publication of the study.

## Conflicts of Interest

The authors declare no conflicts of interest.

## Supporting information


**Video S1.** The video showed the main steps of robotic segmentectomy, the B6 fistula, and the intraoperative management of bronchial defect.

## Data Availability

The clinical data were available on request due to privacy.
